# Risk of Tuberculosis in Children with Juvenile Idiopathic Arthritis: A Nationwide Population-Based Study in Taiwan

**DOI:** 10.1371/journal.pone.0128768

**Published:** 2015-06-05

**Authors:** Yi-Chen Hsin, Lai-Zhen Zhuang, Kuo-Wei Yeh, Cheng-Wei Chang, Jorng-Tzong Horng, Jing-Long Huang

**Affiliations:** 1 Department of Pediatric Allergy Immunology and Rheumatology, Division of Pediatrics, Chang Gung Memorial Hospital, Taoyuan, Taiwan; 2 Department of Computer Science and Information Engineering, National Central University, Chungli, Taiwan; 3 Department of Information Management, Hsing Wu University, New Taipei City, Taiwan; 4 Department of Biomedical Informatics, Asia University, Taichung, Taiwan; Glaxo Smith Kline, DENMARK

## Abstract

**Objective:**

We aimed to determine the risk of tuberculosis in children with juvenile idiopathic arthritis (JIA) in Taiwan.

**Methods:**

We used the Taiwan National Health Insurance Research Database (NHIRD) to conduct a nested case-control study. We identified a JIA cohort and matched each JIA child with non-JIA children for comparison. Methotrexate (MTX), tumor necrosis factor (TNF) inhibitor administration, and new tuberculosis cases were determined during our study period. To compare tuberculosis (TB) risk among our study groups, Cox proportional regression models were used to determine adjusted hazard ratios (aHRs).

**Results:**

We identified 1495 children with JIA and 11592 non-JIA children. Majority (68.7%) children with JIA had not received MTX or TNF inhibitors; 23.9% used MTX without TNF inhibitors, and 7.4% received TNF inhibitors, irrespective of MTX administration. In total, 43 children developed tuberculosis. The overall tuberculosis infection rate for children with JIA was two times higher than that for non-JIA children. Compared with non-JIA children, children with JIA who used MTX without TNF inhibitors revealed a significantly increased of tuberculosis infection rate (aHR = 4.67; 95% CI: 1.65–13.17; P = 0.004). Children with JIA who either received TNF inhibitors or never used MTX and TNF inhibitors revealed a tuberculosis infection rate comparable to that of non-JIA children.

**Conclusions:**

Analysis of nationwide data of Taiwan suggested that children with JIA were at higher risk of tuberculosis compared with those without JIA.

## Introduction

Anti-tumor necrosis factor (TNF) therapy was a breakthrough in managing juvenile idiopathic arthritis (JIA). However, population-based studies have indicated that TNF inhibitors increase the risk of tuberculosis (TB) for adults with rheumatoid arthritis (RA) [1–4]. Despite the extensive use of biologics in pediatrics, the relationship between TB and JIA remains unclear, particularly in TB-endemic areas. Therefore, effects of JIA therapy on TB development require more thorough investigation.

JIA is the most common pediatric rheumatic disease, with an incidence of 3.80–4.93 per 100,000 in Taiwan [5, 6]. JIA leads to morbidities such as joint deformities, uveitis, and altered lipid profiles and increases the risk of cardiovascular diseases [7, 8]. Some reports have documented that JIA remains active into adulthood and results in disabilities [9–11]. Although medical advances have attempted to improve outcomes of JIA, infections, particularly TB, remain a major concern for pediatric rheumatologists.

In 2012, TB infected 8.6 million individuals and resulted in 1.3 million deaths worldwide [12]. Patients with chronic rheumatic diseases who received immunosuppressive treatments were at a higher risk of TB infection or reactivation of a latent TB infection. Most of these findings were based on adults with rheumatoid arthritis and in countries with low TB prevalence [2, 13]. However, very few studies have focused on JIA or on regions with intermediate to high TB prevalence.

Therefore, we conducted a nationwide retrospective nested case-control study to evaluate the risk of TB for pediatric patients with JIA in an area of intermediate TB prevalence in Taiwan. To our knowledge, this is the first study to address this issue in an Asian population.

## Materials and Methods

### Data Source

This study was approved by the Institutional Review Board of the Chang Gang Memorial Hospital (103-5613B). Our data were obtained from the Taiwan National Health Insurance Research Database (NHIRD). This computerized database was derived from the Taiwan National Health Insurance Program and was managed by the Taiwan National Health Research Institute. The Taiwan National Health Insurance Program was established in 1995. This system provides universal health coverage and equal medical access to all Taiwan citizens. In 2011, the coverage rate of the National Health Insurance in Taiwan was 99.6%. Thus, almost the entire population of Taiwan (23 million) was enrolled in this program. NHIRD included patient demographic information, encrypted identification numbers, gender, birth dates, admission dates, diagnostic data and procedures, dates of diagnosis, dates of medical treatment, International Classification of Diseases, Ninth Revision, Clinical Modification (ICD-9-CM) diagnosis codes, and drug codes.

### Study Population

We conducted a nested case-control study via NHIRD. Using NHIRD from 2003 to 2005, two nation-wide cohorts were identified on the basis of diagnosis codes: JIA and non-JIA. The JIA cohort for our study included children younger than 16 years of age with two or more JIA physician diagnosis codes that were at least 7 days but not more than 183 days apart. In addition, these children had pharmacy claims associated with JIA such as nonsteroidal anti-inflammatory drugs (NSAIDs), methotrexate (MTX), or TNF inhibitors. JIA diagnosis codes included rheumatoid arthritis (ICD-9: 714), psoriatic arthritis (ICD-9: 696.0), ankylosing spondylitis (ICD-9: 720), and inflammatory bowel disease associated arthritis (ICD-9: 713.1), with a concurrent code of 555 or 556. We excluded children with any physician-diagnosed ICD-9 code for organ transplantation, insulin-dependent diabetes mellitus, chronic renal failure, or human immunodeficiency virus infection.

For comparison, a non-JIA cohort was identified from among children younger than 16 years of age and without JIA diagnosis codes. Each child with JIA was matched to non-JIA children on the basis of age, gender, duration of enrollment, and cohort entry date. All children in our cohorts were followed up until TB occurred or until 2010.

### Medication use

MTX and TNF inhibitor administration was determined from pharmacy claims. On the basis of their therapies, we categorized patients with JIA into three groups: the MTX group included patients who used MTX without TNF inhibitors; the TNF inhibitor group included patients who received TNF inhibitors irrespective of MTX; and the unexposed group included patients who never used TNF inhibitors or MTX.

### Outcome Measurement

As outcomes, we focused on the development of a Mycobacterium tuberculosis infection. During our study period, we determined new cases of physician-diagnosed TB. Diagnosis codes for TB included ICD-9: 010–018. Children with physician-diagnosed TB prior to the index date were excluded.

### Statistical Analysis

Chi-square tests were used to compare baseline characteristics among our cohorts. We used Cox proportional regression models to compare TB infection rates and to determine adjusted hazard ratios (aHRs) adjusted for patient age and gender. The non-JIA group was used as a reference. A P-value of <0.05 was considered significant.

## Results

Table 1 presents baseline characteristics of our study cohorts. Using NHIRD, 1495 children with JIA (866 males; 629 females) and 11592 non-JIA children (6488 males; 5104 females) were identified during the period of 2003–2005. The maximum follow-up period was eight years. The JIA cohort comprised three groups on the basis of therapies used: the MTX group (23.9%) included patients who used MTX without TNF inhibitors; the TNF inhibitor group (7.4%) included those who received TNF inhibitors irrespective of MTX use; and the unexposed group (68.7%) included patients who never used MTX and TNF inhibitors. Significant differences were observed for age and the number of follow-up years among these groups (P < 0.001).

**Table 1:** Baseline characteristics of the study cohort.

	JIA cohort (n = 1495)			Non-JIA cohort	
	Methotrexate group	TNF inhibitor group	Unexposed group	(n = 11592)	P value
Total, number (%)	357 (23.9)	111 (7.4)	1027 (68.7)	11592	
Age, mean ± SD years	11.2 ± 3.2	10.5 ± 3.6	12.2 ± 3.3	10.9 ± 3.7	<0.001
Age 0–5 years, number (%)	20 (5.6)	12 (10.8)	56 (5.4)	944 (8.1)	
Age 6–10 years, number (%)	109 (30.5)	38 (34.2)	188 (18.3)	2808 (24.2)	
Age 11–15 years, number (%)	228 (63.9)	61 (55.0)	783 (76.2)	7840 (67.6)	
Females, number (%)	145 (41)	43 (39)	441 (43)	5104 (44)	0.41
Males, number (%)	212 (59)	68 (61)	586 (57)	6488 (56)	
Follow-up, mean ± SD years	5.78 ± 2.23	3.49 ± 1.79	6.57 ± 1.09	7.77 ± 0.61	<0.001

JIA: juvenile idiopathic arthritis; TNF: tumor necrosis factor

Methotrexate group included patients with JIA who used methotrexate without TNF inhibitors.

TNF inhibitor group included patients with JIA who received TNF inhibitors, irrespective of methotrexate use; 104 patients (93.7%) previously or currently used methotrexate.

Unexposed group included patients with JIA who never received methotrexate or a TNF inhibitor.

During our study period, 43 new TB cases were identified among our cohorts: 9 patients with JIA and 34 children without JIA. Among 9 patients with JIA and TB, four used MTX without TNF inhibitors, one received TNF inhibitors, and four never used MTX or TNF inhibitors. Overall, the crude TB infection rate was two times higher among patients with JIA (6.02 per 1000 patients with JIA) compared with that among children without JIA (2.93 per 1000 non-JIA children; Table 2).

**Table 2:** Tuberculosis infection rates by disease cohort and medication use.

	Total number	Number of TB infection	Rate of TB infection (%)
JIA cohort	1495	9	0.60
MTX use without TNF inhibitors	357	4	1.12
TNF inhibitors use, irrespective of MTX	111	1	0.90
Unexposed to MTX and TNF inhibitors	1027	4	0.39
Non-JIA cohort	11592	34	0.29

TB: tuberculosis; JIA: juvenile idiopathic arthritis; MTX: methotrexate; TNF: tumor necrosis factor

We used Cox proportional hazard regression models to determine aHR for developing TB infection for each group after adjusting for age and gender. Compared with the non-JIA cohort, JIA patients who used MTX without TNF inhibitors revealed a markedly increased TB infection rate (aHR = 4.67; 95% CI: 1.65–13.17; P = 0.004). Further, patients with JIA who received TNF inhibitors and were not exposed to MTX and TNF inhibitors did not present significantly increased TB infection rates (TNF inhibitor group vs. non-JIA cohort: aHR = 5.43; 95% CI: 0.73–40.18; P = 0.097; unexposed group vs. non-JIA cohort: aHR = 1.5; 95% CI: 0.53–4.25; P = 0.445; Table 3).

**Table 3:** Hazard ratios for tuberculosis infection rates.

JIA group by medication exposure	Reference group	Adjusted hazard ratio* (95% CI)	p-value
MTX use without TNF inhibitor	Non-JIA cohort	4.67 (1.65–13.17)	0.004
TNF inhibitor use, irrespective of MTX	Non-JIA cohort	5.43 (0.73–40.18)	0.097
Unexposed to MTX and TNF inhibitor	Non-JIA cohort	1.50 (0.53–4.25)	0.445

JIA: juvenile idiopathic arthritis; 95% CI: 95% confidence interval; MTX: methotrexate; TNF: tumor necrosis factor

* Adjusted for patient age and gender

## Discussion

To our knowledge, this is probably the first study focused on an Asian population to evaluate the risk of TB development in children with JIA in an area with an intermediate TB prevalence. Our nested case-control study identified that the TB infection rate for children with JIA was two times compared with that for children without JIA in Taiwan. This was in contrast to another population-based study conducted in the United States (US). Using the US Medicaid administrative claims data, Beukelman et al. reported that children with JIA did not present a higher TB infection rate compared with those with attention-deficit hyperactivity disorder (ADHD) [14]. One reason for these different results may have been follow-up periods. The median follow-up period in the study by Beukelman et al. was 1.2 years. However, evaluation of the risk of TB among JIA children requires long-term observation. Besides, geographic regions and ethnic differences may influence these results. Taiwan is a TB-endemic area, with a TB incidence of 54.5 per 100,000, as reported in 2011[15], whereas US is a region where TB is under control, with a TB incidence of 3.2 cases per 100,000, as reported in 2012. The major ethnicity observed in our study was Chinese, whereas in the study by Beukelman et al., the major ethnicity observed was Caucasians.

In addition, we examined effects of immunosuppressive agents on TB development. We could not determine any strong association between TB and TNF inhibitor use for children with JIA. It is well known, TNF blockers pose an increased risk of TB infection in adults with rheumatoid arthritis. This evidence was derived from animal studies and from population-based observations in countries with low TB prevalence, including US, Sweden, France, and the United Kingdom [1–4, 16]. However, there have been very few well-designed population-based studies for children with JIA.

Pediatric data regarding TNF inhibitor use have primarily derived from clinical trials and large registries. Very few TB cases have been reported among pediatric patients under anti-TNF therapy [17–20]. Our nationwide study provided the first population-based data. Although the TB case number was limited in the anti-TNF group, it suggested that TNF inhibitors did not significantly increase the risk of TB among children with JIA, even in an area with an intermediate incidence of TB. There may be several reasons for this.

First, a majority of anti-TNF therapy for JIA in Taiwan comprises the use of etanercept, which is associated with a lower risk of TB. The risk of TB varies with different anti-TNF agents. Adalimumab or infliximab use revealed a three- to four-fold higher risk of TB when compared with entanercept for patients with rheumatoid arthritis [21].

Second, TNF inhibitors have better disease activity control and require lower corticosteroid doses. TNF inhibitors may decrease the risk of TB through a steroid-sparing effect. A clinical study in Finland that evaluated effects of anti-TNF therapy on the growth of patients with severe JIA revealed significantly decreased corticosteroid doses during anti-TNF therapy [22]. Kou et al. reported that not only prednisolone but also MTX and other immunosuppressive drugs were successfully tapered after a mean of 2.5 months of etanercept therapy for patients with refractory JIA in Taiwan [23].

Third, TB prevalence is relatively low among children than adults. Bacillus Calmette-Guerin vaccination is mandatory in Taiwan, with a coverage rate of approximately 98%. Routine Bacillus Calmette-Guerin vaccination prevents younger children from developing tuberculosis; therefore, the childhood TB incidence was 9.61 per 100,000 patient-years in Taiwan, which was much lower compared with that in adults [24]. In addition, routine latent TB screening and careful selection of candidates before initiating anti-TNF therapy has helped to decrease TB development according to experiences reported in Western countries [2]. These data supported the assumption that the risk of TB varied in association with TNF inhibitors between adults with rheumatoid arthritis and children with JIA.

Moreover, our results indicated that patients with JIA who used MTX without TNF inhibitors were at a higher risk for TB when compared with non-JIA children (aHR = 4.67; 95% CI: 1.65–13.17; P = 0.004). Our finding was similar to that from a rheumatoid arthritis cohort in Quebec, Canada; they identified 50 TB cases from among a rheumatoid arthritis cohort of 24,282 patients. The adjusted rate ratio of TB was 3.0 (95% CI: 1.6–5.8) with nonbiologic DMARD use [25]. Therefore, when treating patients with JIA, we should not underestimate the MTX-associated risk of TB.

The strength of our study was the provision of a nationwide population-based study of Asian children with JIA regarding the risk of developing TB in a TB-endemic area. However, there were several limitations. NHIRD did not provide detailed information such as JIA subtypes, disease activity, and contact history of TB, which were possible confounding factors for this study. In addition, we experienced difficulties with obtaining accurate information regarding medications used. Body weights of patients and inpatient medications were missing in NHIRD; therefore, we could not calculate actual medication duration and doses. For example, we could not calculate corticosteroid doses (mg/kg/day).

In conclusion, this nationwide study of 1495 patients with JIA with long-term follow-ups indicated that the TB infection rate among children with JIA was two times higher compared with that among children without JIA.
